# 双药方案对比单药方案治疗老年晚期非小细胞肺癌的*Meta*分析

**DOI:** 10.3779/j.issn.1009-3419.2012.06.07

**Published:** 2012-06-20

**Authors:** 崇安 许, 子又 昌, 小杰 王, 琳 李, 海燕 齐, 艳 刘

**Affiliations:** 110032 沈阳，中国医科大学附属第四医院肿瘤内科 Department of Oncology, the Fourth Affiliated Hospital, China Medical University, Shenyang 110032, China

**Keywords:** 肺肿瘤, 老年, 化疗, *Meta*分析, Lung neoplasms, Aged, Chemotherapy, *Meta*-analysis

## Abstract

**背景与目的:**

双药方案治疗老年晚期非小细胞肺癌（non-small cell lung cancer, NSCLC）的疗效是否优于单药化疗尚存争议，本研究旨在对双药方案治疗老年晚期NSCLC患者的有效性和安全性进行系统评价。

**方法:**

计算机检索PubMed、EMBASE、Cochrane Library、中国期刊全文数据库和中国生物医学文献等数据库，收集双药方案治疗老年晚期NSCLC的随机对照试验，用Stata 11.0软件对数据进行*meta*分析。

**结果:**

共纳入12项随机对照试验（2, 306例病例），*meta*分析结果显示与单药化疗相比双药化疗明显提高了老年晚期NSCLC患者的有效率（OR=1.80, 95%CI: 1.50-2.17, *P* < 0.000, 1）和1年生存率（OR=1.45, 95%CI:1.22-1.72, *P* < 0.000, 1）；含铂双药（OR=1.55, 95%CI:1.18-2.03, *P*=0.001）和非铂双药组（OR=1.38, 95%CI: 1.10-1.73, *P*=0.006）的1年生存率均明显高于单药组；含铂双药组更易发生3/4级贫血、中性粒细胞减少、血小板减少和神经毒性（*P* < 0.05），非铂双药组毒副反应发生率与单药组相似。

**结论:**

与单药组相比，双药组可明显提高化疗有效率和生存率，更适合作为老年晚期NSCLC一线化疗方案，但尚需开展针对老年患者的随机对照试验加以验证。

## Abstract (en)

**Background and objective:**

It remains disputed whether doublets are more effective than single-agent chemotherapy for elderly patients with advanced non-small cell lung cancer (NSCLC). The aim of this study is to evaluate the efficacy and safety of doublets and single-agent chemotherapy for elderly patients with NSCLC.

**Methods:**

Data from all published, randomized trials that compared doublets and single-agent chemotherapy in elderly patients were collected from electronic databases (PubMed, EMBASE, Cochrane Library, CNKI and the CBMdice). *Meta*-analysis was completed using software Stata 11.0.

**Results:**

The results of the *meta*-analysis, including 12 eligible trials (2, 306 patients), showed that the doublets significantly increased the overall response rate (OR=1.80, 95%CI:1.50-2.17, *P* < 0.000, 1) and one-year survival rate (OR=1.45, 95%CI: 1.22-1.72, *P* < 0.000, 1) compared with single-agent chemotherapy. The results of one-year survival rate in platinum-based doublet chemotherapy arms (OR=1.55, 95%CI: 1.18-2.03, *P*=0.001) and non platinum-based ones (OR=1.38, 95%CI: 1.10-1.73, *P*=0.006) were both significantly higher than that of single-agent chemotherapy. However, grade 3/4 anemia, neutropenia, thrombocytopenia and neurotoxicity (*P* < 0.05) were significantly associated with doublet chemotherapy. The incidence of toxicity effect in non platinum-based chemotherapy was similar to that of single-agent chemotherapy.

**Conclusion:**

Compared with single-agent chemotherapy, doublet chemotherapy could increase the overall response rate and one-year survival rate significantly. Therefore, doublet chemotherapy would be more appropriate for elderly patients with advanced NSCLC as the first-line chemotherapy regimen. However, further prospective randomized controlled trials in elderly NSCLC patients is needed to verify the findings in this study.

随着人口预期寿命的大幅度提高，其患癌症风险亦随之增加，导致老年人肺癌发病率明显上升^[1]^。在被确诊的非小细胞肺癌（non-small cell lung cancer, NSCLC）中年龄超过65岁的比例超过50%，其中70岁以上者占30%-40%^[2, 3]^。因此针对这一群体的治疗凸显重要。临床研究^[4, 5]^表明含铂双药方案优于单药或三药联合方案，但由于纳入和排除标准等限制，老年肺癌在临床试验中的代表人数不足。因此，含铂双药方案仅被证实是适合非老年的晚期NSCLC患者的标准一线化疗方案^[6, 7]^，而老年晚期NSCLC最佳治疗方案仍在争议中。

目前的证据^[8-10]^表明，与最佳支持治疗相比第三代化疗药物（长春瑞滨、吉西他滨、紫杉醇和多西紫杉醇）单药化疗不但能够延长老年晚期NSCLC患者的生存期，还能提高其生活质量。因此，多数老年晚期NSCLC患者首选第三代化疗药物单药化疗。但也有研究^[11-13]^表明双药方案联合化疗对老年晚期NSCLC患者同样有效，且毒性可耐受。为明确双药方案与单药方案在治疗老年晚期NSCLC患者中哪一种更具优越性，本研究运用Cochrane系统评价的方法比较两者在有效性和安全性方面的差异，以期为临床决策提供参考依据。

## 1 材料与方法

### 1.1 纳入与排除标准

#### 1.1.1 研究设计

随机对照试验（radonmized controlled trails, RCTs），无论是否采用盲法。

#### 1.1.2 研究对象

纳入标准：①病理/经细胞学证实的Ⅲb期-Ⅳ期NSCLC；②既往未接受过其它抗肿瘤治疗并且无化疗禁忌症；③美国东部肿瘤协作组-体力状况（Eastern Cooperative Oncology Group-performance status, ECOG-PS）评分≤2分；④年龄≥65岁。排除标准：①伴有严重内科疾病及感染；②同时伴随其它恶性肿瘤；③肺癌为其它肿瘤转移病灶；④动物实验和体外试验。

#### 1.1.3 干预措施

试验组采用以第三代化疗药物为基础的双药方案化疗，对照组采用单药化疗。

#### 1.1.4 结局指标

1年生存率（化疗后仍然存活1年的患者比例）；化疗有效率（objective response rate, ORR），ORR是指化疗2个周期后按照WHO或RECIST标准达到完全和部分缓解所占的比例；毒副反应，包括血液学毒性和胃肠道反应及神经毒性。

### 1.2 检索策略

计算机检索PubMed、EMBASE、Cochrane Library、中国期刊全文数据库（CNKI）、中国生物医学文献数据库（CBMdice），检索时间从建库至2011年11月。检索词包括：non-small cell lung cancer、non small cell lung cancer、non small cell lung carcinoma、non small cell lung carcinomas、non small cell lung、NSCLC、malignant epithelial tumor、elderly、pharmacotherapy、antineoplastic combined chemotherapy、非小细胞肺癌、老年、药物治疗、抗肿瘤药物联合化疗等。RCTs检索策略遵循Cochrane系统评价手册5.0，其它检索采用主题词与自由词结合的方式，并根据具体数据库调整，所有检索策略通过多次预检索后确定。同时通过手工搜索相关书籍，挑选相关文章和已发表的文章来补充结果。追查已纳入文献的参考文献，与本领域的专家、通信作者等联系，以获取以上检索未发现的相关信息。当1个会议的摘要与1篇全文都提到了相同的试验时，只评估全文。当2个或更多文章报道相同的数据时，只评估最近、最新的数据。文献语种限中文和英文。

### 1.3 文献筛选和资料提取

两名研究者交叉核对纳入研究的结果，对有分歧者通过讨论或由第三名研究者仲裁解决。提取的信息资料主要包括：第一作者姓名、发表的杂志和日期、患者的年龄、使用的药物和剂量、患者的数目、1年生存率、ORR以及发生3/4级毒副反应患者的比例。

### 1.4 文献质量评价

采用Cochrane Handbook 5.0对纳入研究进行方法学质量评价，内容包括：①采用何种随机方法，方法是否正确；②是否进行分配隐藏，方法是否正确；③是否采用盲法，对哪些病例实施了盲法；④有无失访和退出，是否采用意向性（intent-to treat, ITT）分析。依据评价结果，将纳入文献分为A、B、C三个等级。

### 1.5 统计学分析

采用Stata 11.0软件进行数据分析。双药化疗组作为试验组，单药化疗组作为对照组。在meta分析中如果1个试验中有2个以上不同化疗方案的比较，则分别给试验组或对照组的数目记为2次或更多次，因此，实际上比较的组数要多于包含的试验数目。计数资料采用比值比（odds ratio, OR）为疗效分析统计量，各效应量均以95%CI表示。各纳入研究结果间的异质性采用*χ*^2^检验。若*P*>0.1和*I*^2^ < 50%时，采用固定效应模型进行分析；若存在统计学异质性（*P* < 0.1, *I*^2^ > 50%）时，分析异质性来源，确定是否能采用随机效应模型。如果研究间存在明显的临床异质性，只对其进行描述性分析。必要时采用敏感性分析检验结果的稳定性。

## 2 结果

### 2.1 检索结果

按照检索策略和资料收集方法（图 1）共查到相关文献1, 295篇，通过排除重复、阅读文题、摘要和全文后最终纳入12项RCTs研究^[14-25]^。所有的数据均从纳入试验中提取。在这些试验中共有2, 306例患者，1, 055例患者接受了以第三代化疗为基础的双药方案化疗，1, 251例患者接受了单药方案化疗。12项试验中有3项试验最低年龄限制是65岁^[18, 19, 22]^，其余9项试验最低年龄限制都是70岁。因为有3项试验有多于2个的对比组^[15, 16, 18]^，因此进行对比的组数共有17组。纳入研究的一般特征见表 1。

**1:**
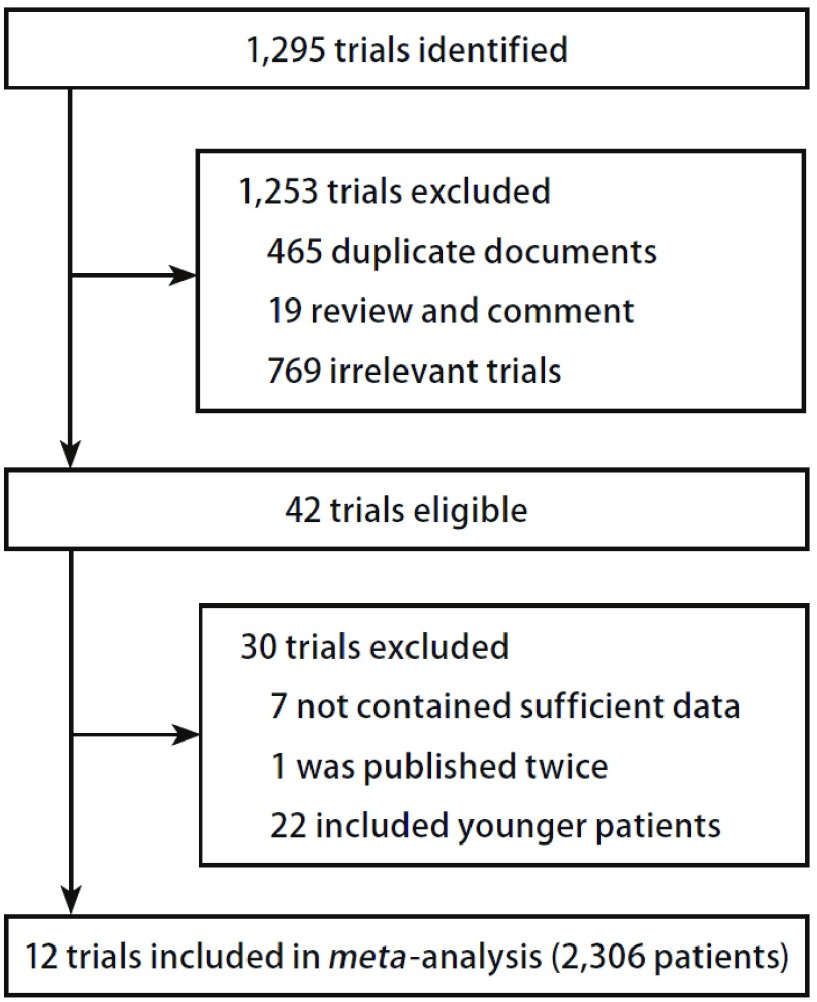
纳入研究流程图 Trials enrolled in the study

**1:** 纳入研究的一般特征 The characteristics of included

Reference	Arm	Treatment schedule and dose	No. of patients	Age (yr)	PS	OS (wk)	1-y SR (%)	ORR (%)
Frasci 2001^[14]^ (Lung Cancer)	Traetment	Gemcitabine at 1, 200 mg/m^2^ on days 1 and 8, every 3 weeks Vinorelbine at 30 mg/m^2^ on days 1 and 8, every 3 weeks	60	≥70	16	29	30	22
	Control	Vinorelbine at 30 mg/m^2^ on days 1 and 8, every 3 weeks	60	≥70	13	18	13	15
Gridelli 2003^[15]^ (J Natl Cancer Inst)	Traetment	Gemcitabine at 1, 000 mg/m^2^ on days 1 and 8, every 3 weeks Vinorelbine at 25 mg/m^2^ on days 1 and 8, every 3 weeks	232	≥70	44	30	30	21
	Control	Gemcitabine at 1, 200 mg/m^2^ on days 1 and 8, every 3 weeks	233	≥70	41	28	28	16
	Traetment	Gemcitabine at 1, 000 mg/m^2^ on days 1 and 8, every 3 weeks Vinorelbine at 25 mg/m^2^ on days 1 and 8, every 3 weeks	232	≥70	44	30	30	21
	Control	Vinorelbine at 30 mg/m^2^ on days 1 and 8, every 3 weeks	233	≥70	45	36	38	18
Comella 2004^[16]^ (Br J Cancer)	Traetment	Gemcitabine at 1, 000→1, 200 mg/m^2^ on days 1 and 8, every 3 weeks Vinorelbine at 25→30 mg/m^2^ on days 1 and 8, every 3 weeks	68	≥70	21	9.7	32	23
	Control	Gemcitabine at 1, 200→1, 400→1, 600 mg/m^2^ on days 1 and 8 and 15, every 4 weeks	68	≥70	19	5.1	29	18
	Traetment	Gemcitabine at 1, 000→1, 200 mg/m^2^ on days 1 and 8, every 3 weeks Vinorelbine at 25→30 mg/m^2^ on days 1 and 8, every 3 weeks	68	≥70	21	9.7	32	23
	Control	Paclitaxei at 100→120→140 mg/m^2^ on days 1, 8 and 15, every 4 weeks	63	≥70	22	6.4	25	13
	Traetment	Gemcitabine at 1, 000→1, 200 mg/m^2^ on days 1 and 8, every 3 weeks Paclitaxei at 80→100 mg/m^2^ on days 1 and 8, every 3 weeks	65	≥70	15	9.2	44	32
	Control	Gemcitabine at 1, 200→1, 400→1, 600 mg/m^2^ on days 1, 8 and 15, every 4 weeks	68	≥70	19	5.1	29	18
	Traetment	Gemcitabine at 1, 000→1, 200 mg/m^2^ on days 1 and 8, every 3 weeks Paclitaxei at 80→100 mg/m^2^ on days 1 and 8, every 3 weeks	65	≥70	15	9.2	44	32
	Control	Paclitaxei at 100→120→140 mg/m^2^ on days 1, 8 and 15 every 4 weeks	63	≥70	22	5.1	25	13
Sun Q 2006^[17]^ (Chin J Geriatr)	Traetment	Gemcitabine at 1, 000 mg/m^2^ on days 1 and 8, every 3 weeks Cisplatin at 20 mg/m^2^ on days 1 to 3, every 3 weeks	22	≥70	8	9.2	45.5	40.9
	Control	Vinorelbine at 25 mg/m^2^ on days 1 and 8, every 3 weeks	23	≥70	9	8.8	43.5	34.9
Zhang K 2006^[18]^ (Chin J Clin Oncol)	Traetment	Paclitaxel at 60 mg/m^2^ on days 1, 8 and 15, every 4 weeks Cisplatin at 30 mg/m^2^ on days 2 to 4, every 4 weeks	34	≥65	-	9	38.2	55.9
	Control	Paclitaxel at 60 mg/m^2^ on days 1, 8 and 15, every 4 weeks	30	≥65	-	8	36.7	26.7
	Traetment	Paclitaxel at 60 mg/m^2^ on days 1, 8 and 15, every 4 weeks Carboplatin at 5 AUC on days 2, every 4 weeks	32	≥65	-	10	43.8	56.3
	Control	Paclitaxel at 60 mg/m^2^ on days 1, 8 and 15, every 4 weeks	30	≥65	-	8	36.7	26.7
Hainsworth 2007^[19]^ (Cancer)	Traetment	Docetaxel at 30 mg/m^2^ on days 1, 8 and 15, every 4 weeks Gemcitabine at 800 mg/m^2^ on days 1 and 8 and 15, every 4 weeks	174	> 65	65	5.5	26	25 (126)
	Control	Docetaxel at 36 mg/m^2^ on days 1, 8 and 15, every 4 weeks	171	> 65	57	5.1	24	17 (130)
Huang C 2007^[20]^ (J Tianjin Med University)	Traetment Control	Vinorelbine at 25 mg/m^2^ on days 1 and 5, every 3 weeks Cisplatin at 20 mg/m^2^ on days 2 to 4, every 3 weeks Vinorelbine at 25 mg/m^2^ on days 1 and 5, every 3 weeks	28 30	≥70 ≥70	- -	9.3 9.1	46.4 43.3	39.3 30
Chen 2008^[21]^ (Lung Cancer)	Traetment	Vinorelbine at 22.5 mg/m^2^ on days 1 and 8, every 3 weeks Cisplatin at 50 mg/m^2^ on days 1, every 3 weeks	34	≥70	16	11.3	47.2	32.4
	Control	Vinorelbine at 25 mg/m^2^ on days 1 and 8, every 3 weeks	31	≥70	16	12	50.9	16.1
Li PY 2008^[22]^ (J Clin Pulmonary Med)	Traetment	Paclitaxel at 135 mg/m^2^ on days 1, every 3 weeks Cisplatin at 20 mg/m^2^ on days 2 to 4, every 3 weeks	29	≥65	-	-	46.4	37.9
	Control	Paclitaxel at 135 mg/m^2^ on days 1, every 3 weeks	29	≥65	-	-	43.3	31
Jiang JL 2010^[23]^ (Clin J Traditional Med)	Traetment	Paclitaxel at 175 mg/m^2^ on days 1, every 3 weeks Oxaliplatin at 130 mg/m^2^ on days 1, every 3 weeks	18	≥70	16	11.4	50	44.4
	Control	Paclitaxel at 175 mg/m^2^ on days 1, every 3 weeks	20	≥70	16	13.2	44	35
Lou GY 2010^[24]^ (Natl Med J China)	Traetment	Gemcitabine at 1, 000 mg/m^2^ on days 1 and 8, every 3 weeks Carboplatin at 5 AUC mg/m^2^ on days 2, every 3 weeks	34	≥70	3	-	32	41
	Control	Gemcitabine at 1, 000 mg/m^2^ on days 1 and 8, every 3 weeks	34	≥70	3	-	31	38
Quoix 2011^[25]^ (Lancet)	Traetment	Paclitaxei at 90 mg/m^2^ on day 1, 8 and 15, every 4 weeks Carboplatin at 6 AUC on day 1, every 4 weeks	225	≥70	61	10.3	44.5	27.1
	Control	Gemcitabine at 1, 150 mg/m^2^ on days 1 and 8, every 3 weeks & Vinorelbine at 25 mg/m^2^ on days 1 and 8, every 3 weeks	226	≥70	62	6.2	25.4	10.2
→: dose escalation; PS: performance status; OS: overall survival; 1-y SR: 1-year survival rate; ORR: overall response rate.								

### 2.2 纳入研究质量评价

9项研究的质量被评为“B”级，3项研究的质量被评为“C”级（表 2），尽管所有研究均未提及盲法，且仅1项研究提及分配隐藏，但不会对本研究主要结局指标（1年生存率）产生影响，因此，本研究可信度较高。

**2:** 纳入研究的方法学质量 Quality assessment of methodology of included studies

Included studies	Randomization	Allocation concealment	Blinding	Lost to follow up	ITT Analysis	Baseline	Quality grading
Frasci 2001^[14]^	Unclear	Unclear	Unclear	Yes	Yes	Similar	B
Gredelli 2003^[15]^	Stratified	Unclear	Unclear	Yes	Yes	Similar	B
Comella 2004^[16]^	Computerized	Unclear	Unclear	Yes	Yesr	Similar	B
Sun Q 2006^[17]^	Numbered	Unclear	Unclear	Yes	Unclear	Unclear	B
Zhang K 2006^[18]^	Unclear	Unclear	Unclear	Yes	Yes	Unclear	B
Hainsworth 2007^[19]^	Unclear	Unclear	Unclear	Yes	Yes	Similar	B
Huang C 2007^[20]^	Unclear	Unclear	Unclear	Yes	Unclear	Unclear	C
Chen 2008^[21]^	Unclear	Yes	Unclear	Yes	Yes	Similar	B
Li PY 2008^[22]^	Unclear	Unclear	Unclear	Yes	Unclear	Unclear	C
Jiang JL 2010^[23]^	Unclear	Unclear	Unclear	Yes	Unclear	Unclear	C
Lou GY 2010^[24]^	Numbered	Unclear	Unclear	Yes	Unclear	Similar	B
Quoix 2011^[25]^	Stratified	Unclear	Unclear	Yes	Yes	Similar	B
ITT: intent to treat.							

### 2.3 有效率

各研究之间无异质性，采用固定效应模型。12项试验的17个对比组的*meta*分析（图 2）表明，与单药化疗相比，双药化疗明显提高了老年晚期NSCLC患者的有效率（OR=1.80, 95%CI: 1.50-2.17, *P* < 0.000, 1）。

**2:**
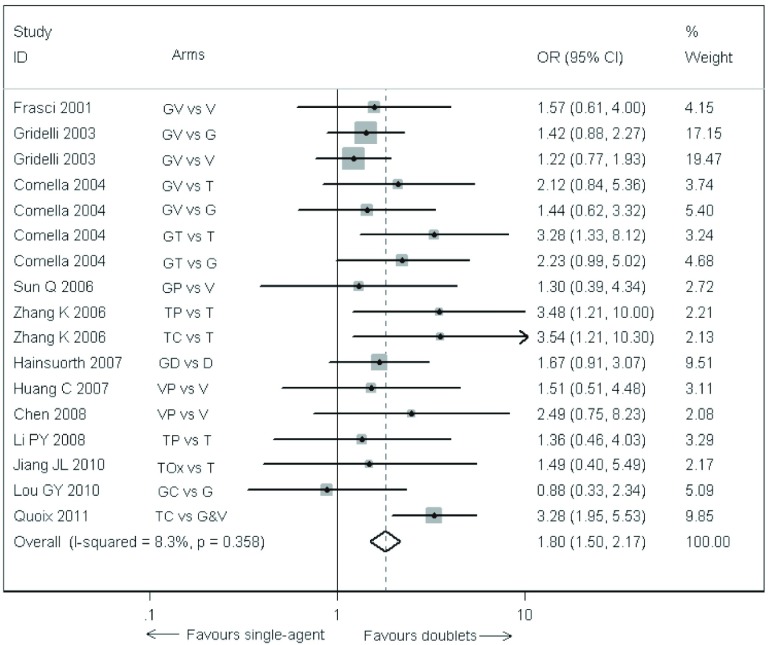
双药方案与单药方案治疗老年晚期非小细胞肺癌患者的有效率。G：吉西他滨；V：长春瑞滨；T：紫杉醇；D：多西紫杉醇；P：顺铂；C：卡铂；Ox：奥沙利铂。 Comparison of the overall response rate between doublet arms and single-agent arms of the elderly patients with advanced non-small cell lung cancer. G: gemcitabine; V: vinorelbine; T: Taxol (paclitaxel); D: docetaxel; P: cisplatin; C: carboplatin; Ox: Oxaliplatin.

### 2.4 生存率

各研究间存在明显的异质性（*P*=0.04, *I*^2^=42%），进一步亚组分析显示8项^[17, 18, 20-25]^含铂双药方案各研究间无异质性（*P*=0.363, *I*^2^=8.6%），采用固定效应模型合并分析显示，与单药化疗相比含铂双药化疗明显提高了老年晚期NSCLC患者的1年生存率（OR=1.55, 95%CI: 1.18-2.03, *P*=0.001）。4项（8个对比组）^[14-16, 19]^非铂双药方案各研究间存在异质性（*P*=0.003, *I*^2^=55.7%），进一步行敏感性分析，将异质性较大的Gridelli等^[15]^研究中的吉西他滨联合长春瑞滨对比长春瑞滨单药组剔除后，不但消除了非铂双药方案的异质性，同时也消除了所有试验其余16个对比组之间的异质性。*meta*分析显示非铂双药方案（OR=1.38, 95%CI:1.10-1.73, *P*=0.006）和所有采用双药方案化疗的老年晚期NSCLC患者的1年生存率均明显高于单药化疗者（OR=1.45, 95%CI:1.22-1.72, *P* < 0.000, 1），见图 3。而Gridelli等^[15]^研究中的吉西他滨联合长春瑞滨组与长春瑞滨单药组的1年生存率相比无统计学差异（OR=0.70, 95%CI: 0.48-1.03, *P*=0.069）。

**3:**
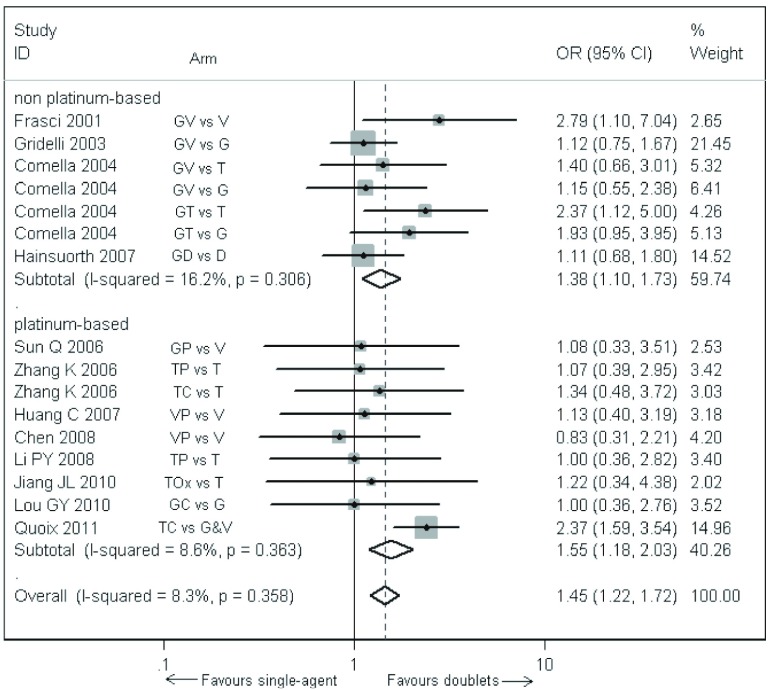
双药方案与单药方案治疗老年晚期非小细胞肺癌患者的1年生存率。G：吉西他滨；V：长春瑞滨；T：紫杉醇；D：多西紫杉醇；P：顺铂；C：卡铂；Ox：奥沙利铂。 Comparison of the 1-year survival rate between doublet arms and single-agent arms of the elderly patients with advanced non-small cell lung cancer. G: gemcitabine; V: vinorelbine; T: Taxol (paclitaxel); D: docetaxel; P: cisplatin; C: carboplatin; Ox: Oxaliplatin.

### 2.5 毒副反应

由于铂类药物的毒副反应与其它药物明显不同，故将含铂方案和非铂方案的毒副反应分组分析。

#### 2.5.1 含铂双药方案的毒副反应

4项试验^[17, 21, 24, 25]^报道了3/4级贫血的发生率，4项试验^[20, 21, 23, 25]^报道了3/4级中性粒细胞减少的发生率，7项试验^[17, 20-25]^报道了3/4级血小板减少的发生率，6项试验^[17, 20-23, 25]^报道了3/4级恶心、呕吐的发生率，4项试验^[20, 21, 23, 25]^报道了3/4级神经毒性的发生率。meta分析结果显示含铂双药方案更易发生3/4级贫血、中性粒细胞减少、血小板减少和神经毒性（表 3）。

**3:** 含铂双药与单药化疗的3/4级毒副反应 Comparison of Grade 3/4 toxicity between platinum-based doublet arms and single-agent arms in trials

Toxicity	No. of Trials	No. of patients		OR	95%CI	*P*
		Doublet arms	Single-agent arms			
Anemia	4	487	484	2.21	1.19-4.13	0.013
Neutropenia	4	303	306	5.51	3.67-8.27	< 0.001
Thrombocytopenia	7	388	392	5.13	2.48-10.60	< 0.001
Nausea & vomiting	6	354	358	7.48	1.00-55.77	0.050
Neurotoxicity	4	303	306	5.5	1.53-19.83	0.009

#### 2.5.2 非铂双药组毒副反应

纳入研究^[14-16, 19]^均报道了3/4级贫血、中性粒细胞减少、血小板减少及恶心、呕吐的发生率，3项研究^[15, 16, 19]^报道了3/4级神经毒性的发生率。*meta*分析结果显示非铂双药化疗组3/4级毒副反应的发生率与单药化疗组相似（表 4）。

**4:** 非铂双药与单药化疗的3/4级毒副反应 Comparison of grade 3/4 toxicity between non platinum-based doublet arms and single-agent arms in trials

Toxicity	No. of trials	No. of patients		OR	95%CI	*P*
		Doublet arms	Single-agent arms			
Anemia	8	932	926	1.13	0.56-2.30	0.728
Neutropenia	8	932	926	1.28	0.78-2.10	0.324
Thrombocytopenia	8	932	926	1.74	0.88-3.43	0.110
Nausea & vomiting	8	932	926	0.99	0.58-1.70	0.980
Neurotoxicity	7	872	866	1.11	0.44-2.82	0.825

### 2.6 发表偏倚

采用漏斗图对纳入文献潜在的发表偏倚进行检验（图 4），漏斗图图形基本对称，提示发表偏倚的可能性较小。

**4:**
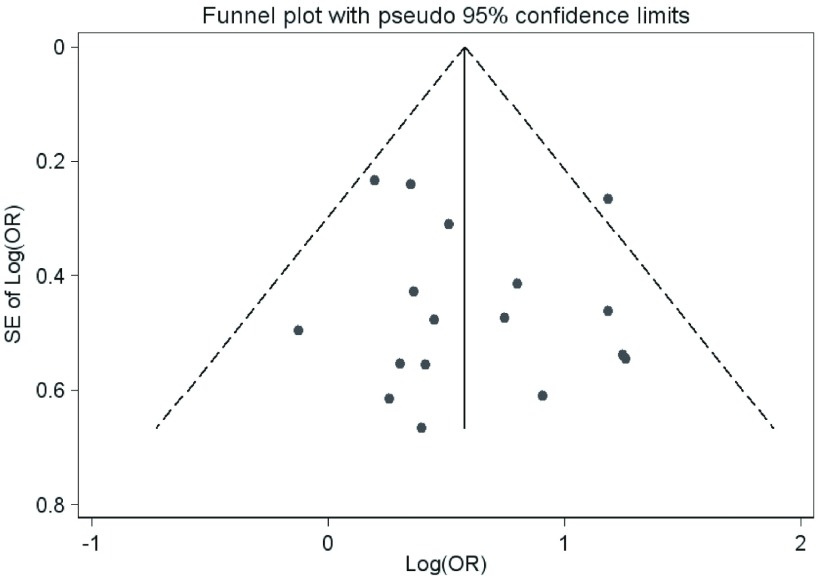
漏斗图 Funnel plot

## 3 讨论

随着年龄的增长，化疗危险性亦随之增加^[26]^，因此，高龄在很多癌症中被当作化疗尤其是联合化疗的绝对或相对禁忌症。但是，由于老年患者是一组异质人群，其生理年龄和实际年龄差异很大，因此单凭年龄并不能决定一个患者是否接受化疗^[27]^。研究^[28-32]^表明老年晚期NSCLC患者不但对单药化疗，甚至对双药化疗也有很好的疗效和耐受性。在包括年龄无上限的老年患者的几项大型随机试验^[30-34]^中，回顾性亚组分析结果显示，采用含铂双药方案化疗的老年晚期NSCLC患者不但肿瘤反应率和总生存与年轻患者相似，而且毒性反应也与年轻患者相似，并没有明显影响老年患者的生活质量。这些数据虽然支持了老年患者使用含铂双药方案化疗的可行性，但是由于他们在试验中的代表人数不足，仅占试验的一小部分，而且是被精心挑选出的能更好耐受化疗的老年群体，并不能代表整个老年人群。因此，双药方案作为老年患者一线优选方案的证据尚不充分，为评价双药方案在治疗老年晚期NSCLC患者中的疗效是否优于单药化疗而进行了本项研究。

研究结果显示，与单药相比双药化疗能够明显提高老年晚期NSCLC患者的有效率。由于各研究间的1年生存率有异质性而进行了亚组分析，结果表明含铂双药各研究间无异质性，合并分析显示与单药化疗相比含铂双药化疗提高了老年晚期NSCLC患者的1年生存率，但也更易发生3/4级血液学和神经毒性。而非铂双药方案各研究间仍存异质性，进一步行敏感性分析发现，剔除异质性较大的Gridelli等^[15]^研究中的吉西他滨联合长春瑞滨对比长春瑞滨单药组后，不但非铂方案之间异质性消除，而且其余16个对比组间的异质性也随之消除。meta分析显示，16个对比组中双药化疗组的1年生存率明显高于单药化疗组；而非铂双药化疗组不但1年生存率明显高于单药化疗组，而且3/4级毒副反应的发生率亦未增加。

尽管本文只纳入了Ⅱ期/Ⅲ期RCTs，但在提取数据中，年龄的异质性导致了患者的选择偏倚。在选定的试验中因有2项试验^[16, 19]^的纳入对象中包含了部分体力状态差的年轻患者，导致了研究人群的异质性。但是，由于体力状态差的年轻患者仅占小部分，而且体力状态差亦预示着预后差^[27, 35]^。因此，这种选择偏倚可能对研究结果的影响甚小。虽然不同化疗方案之间可能存在异质性，但有研究^[36]^表明不同双药方案之间有效率和生存率无统计学差异，因此，不同化疗方案的差异对本研究结果的影响可能较小。

本项研究的质量也受到一些限制，虽然发表偏倚较小，但是由于本项*meta*分析不是基于个体患者资料的数据，而仅仅是从公开发表的文献中提取的数据，因此，治疗效果有可能被过高估计。虽然研究中制定了严格的纳入标准，但各个研究中病例的选择差异、试验设计、药物剂量以及不同药物的联合作用均可产生异质性；并且由于研究结果发表的选择性偏倚，如报道副作用的试验数量少，异质性即使不明显也会显现出来，因此，必须谨慎解释评估的结果。

目前的证据表明，第三代化疗药物单药化疗是非选择的老年晚期NSCLC患者可选择的方案之一，而含铂双药方案作为老年晚期NSCLC患者一线优选方案的证据仍不充分。尽管本项研究显示，含铂双药方案有更高的有效率和生存率，但其毒副反应亦相应增加，因此我们认为可作为体力状态较好患者的选择方案之一；而非铂双药方案不但化疗有效率和1年生存率高于单药化疗组，而且副作用轻微，更适合作为老年晚期NSCLC一线化疗方案。

本次荟萃分析使我们充分认识到，不加选择地将老年晚期NSCLC患者定义为可以或不可以接受单药或双药化疗都是不妥的，应根据老年患者的生理学特性和老年患者的异质性，充分考虑到药物的预期毒性、药代动力学、器官功能和并发症以及患者的意愿进行综合评估化疗的风险/获益比，以使老年晚期NSCLC患者最大获益。今后应进一步开展针对老年晚期NSCLC患者设计的前瞻性随机对比双药与单药的临床试验，为临床决策提供更有说服力的参考依据。

## References

[1] Lebitasy MP, Hédelin G, Purohit A (2001). Progress in the management and outcome of small-cell lung cancer in a French region from 1981 to 1994. Br J Cancer.

[b2] 2Ries LAG, Eisner MP, Kosary CL, et al. SEER Cancer Statistics Review 1975-2000. http://seer.cancer.gov/csr/1975_2000/, 2005-04-07.

[3] Gridelli C, Perrone F, Monfardini S (1997). Lung cancer in the elderly. Eur J Cancer.

[4] Lewis JH, Kilgore ML, Goldman DP (2003). Participation of patients 65 years of age or older in cancer clinical trials. J Clin Oncol.

[5] Hutchins LF, Unger JM, Crowley JJ (1999). Underrepresentation of patients 65 years of age or older in cancer-treatment trials. N Engl J Med.

[6] Pfister DG, Johnson DH, Azzoli CG (2004). American Society of Clinical Oncology treatment of unresectable non-small-cell lung cancer guideline: update 2003. J Clin Oncol.

[7] ESMO (2001). ESMO Minimum Clinical Recommendations for diagnosis, treatment and follow-up of non-small-cell lung cancer (NSCLC). Ann Oncol.

[8] Effects of vinorelbine on quality of life and survival of elderly patients with advanced non-small-cell lung cancer (1999). The Elderly Lung Cancer Vinorelbine Italian Study Group. J Natl Cancer Inst.

[9] Roszkowski K, Pluzanska A, Krzakowski M (2000). A multicenter, randomized, phase Ⅲ study of docetaxel plus best supportive care versus best supportive care in chemotherapy-naive patients with metastatic or non-resectable localized non-small cell lung cancer (NSCLC). Lung Cancer.

[10] Kudoh S, Takeda K, Nakagawa K (2006). Phase Ⅲ study of docetaxel compared with vinorelbine in elderly patients with advanced non-small-cell lung cancer: results of the West Japan Thoracic Oncology Group Trial (WJTOG 9904). J Clin Oncol.

[11] Pujol JL, Milleron B, Molinier O (2006). Weekly paclitaxel combined with monthly carboplatin in elderly patients with advanced non-small cell lung cancer: a multicenter phase Ⅱ study. J Thorac Oncol.

[12] Maestu I, Gómez-Aldaraví L, Torregrosa MD (2003). Gemcitabine and low dose carboplatin in the treatment of elderly patients with advanced non-small cell lung cancer. Lung Cancer.

[13] Tsukada H, Yokoyama A, Nishiwaki Y (2007). Randomized controlled trial comparing docetaxel (D)-cisplatin (P) combination with D alone in elderly patients (pts) with advanced non-small cell lung cancer (NSCLC): JCOG0207. J Clin Oncol.

[14] Frasci G, Lorusso V, Panza N (2001). Gemcitabine plus vinorelbine yields better survival outcome than vinorebine alone in elderly patints with advanced non-small cell lung cancer. A Southern Italy Cooperative Oncology Group (SICOG) phase Ⅲ trial. Lung cancer.

[15] Gridelli C, Perrone F, Gallo C (2003). Chemotherapy for elderly patients with advanced non-small cell lung cancer: the Multicenter Italian Lung Cancer in the Elderly Study (MILES) phase Ⅲ randomized trial. J Natl Cancer Inst.

[16] Comella P, Frasci G, Carnicelli P (2004). Gemcitabine with either paclitaxel or vinorelbine vs paclitaxel or gemcitabine alone for elderly or unfit advanced non-small-cell lung cancer patients. Br J Cancer.

[17] Sun Q, Hua J, Wang Q (2006). A randomized control study of treatment of gemcitabine with or without cisplatin for elderly patients with advanced non-small cell lung cancer. Zhonghua Lao Nian Yi Xue Za Zhi.

[18] Zhang K, Hong J, Xie GR (2006). Weekly Single-agent versus combination chemotherapy for elderly patients with advanced non-small cell lung cancer. Zhongguo Zhong Liu Lin Chuang.

[19] Hainsworth JD, Spigel DR, Farley C (2007). Weekly docetaxel versus docetaxel/gemcitabine in the treatment of elderly or poor performance status patients with advanced nonsmall cell lung cancer: a randomized phase 3 trial of the Minnie Pearl Cancer Research Network. Cancer.

[20] Huang C, Wang X, Li K (2007). A randomized comparative trial of NVB alone versus NVB plus DDP for treatment of elderly patients with non-small cell lung cancer. Tianjin Yi Ke Da Xue Xue Bao.

[21] Chen YM, Perng RP, Shih JF (2008). A phase Ⅱ randomized study of vinorelbine alone or with cisplatin against chemo-naïve inoperable non-small cell lung cancer in the elderly. Lung cancer.

[22] Li PY, Bu BY, Yang JP (2008). Clinical observation of effect of TAX alone versus TAX plus cisplatin for treatment of elderly patients with non-small cell lung cancer. Lin Chuang Fei Ke Za Zhi.

[23] Jiang JL (2010). A comparative trial of paclitaxel plus oxaliplatin-L versus paclitaxel alone for treatment of elderly patients with non-small cell lung cancer. Zhong Yi Yao Lin Chuang Za Zhi.

[24] Lou GY, Li T, Gu CP (2010). Efficacy study of single-agent gemcitabine versus gemcitabine plus carboplatin in untreated elderly patients with stage Ⅲb/Ⅳ non-small cell lung cancer. Zhonghua Yi Xue Za Zhi.

[25] Quoix E, Zalcman G, Oster JP (2011). Carboplatin and weekly paclitaxel double chemotherapy compared with monotherapy in elderly patients with advanced non-small-cell lung cancer: IFCT-0501 randomised, phase 3 trial. Lancet.

[26] Di Maio M, Perrone F, Gallo C (2003). Supportive care in patients with advanced nonsmall-cell lung cancer. Br J Cancer.

[27] Azzoli CG, Baker S Jr, Temin S (2009). American Society of Clinical Oncology clinical practice guideline update on chemotherapy for stage Ⅳ non-small cell lung cancer. J Clin Oncol.

[28] Fidias P, Supko JG, Martins R (2001). A phase Ⅱ study of weekly paclitaxel in elderly patients with advanced non-small cell lung cancer. Clin Cancer Res.

[29] Ricci S, Antonuzzo A, Galli L (2000). Gemcitabine monotherapy in elderly patients with advanced non-small cell lung cancer: a multicenter phase Ⅱ study. Lung Cancer.

[30] Belani CP, Fossella F (2005). Elderly subgroup analysis of a randomized phase Ⅲ study of docetaxel plus platinum combinations versus vinorelbine plus cisplatin for first-line treatment of advanced non small cell lung carcinoma (TAX 326). Cancer.

[31] Langer CJ, Manola J, Bernardo P (2002). Cisplatin-based therapy for elderly patients with advanced non-small-cell lung cancer: implications of Eastern Cooperative Oncology Group 5592, a randomized trial. J Natl Cancer Inst.

[32] Lilenbaum RC, Herndon JE 2nd, List MA (2005). Single-agent versus combination chemotherapy in advanced non-small cell lung cancer: the cancer and leukemia group B (study 9730). J Clin Oncol.

[33] Rocha Lima CMS, Herndon JE 2nd, Kosty M (2002). Therapy choices among older patients with lung carcinoma. Cancer.

[34] Hensing TA, Peterman AH, Schell MJ (2003). The impact of age on toxicity, response rate, quality of life, and survival in patients with advanced, stage ⅢB or Ⅳ nonsmall cell lung carcinoma treated with carboplatin and paclitaxel. Cancer.

[35] Frasci G, Comella P, Panza N (1998). Carboplatin-oral etoposide personalized dosing in elderly non-small cell lung cancer patients. Gruppo Oncologico Cooperativo Sud-Italia. Eur J Cancer.

[36] Fisher MD, D'Orazio A (2000). Phase Ⅱ and Ⅲ trials: comparison of four chemotherapy regimens in advanced non small-cell lung cancer (ECOG 1594). Clin Lung Cancer.

